# Emerging Molecular Insights and Therapeutic Directions in Neurofibromatosis Type 1 and NF2-Related Schwannomatosis

**DOI:** 10.3390/ijms27062867

**Published:** 2026-03-22

**Authors:** Soyoung Park, Tae-Gyun Woo, So-mi Kang, Bae-Hoon Kim, Bum-Joon Park

**Affiliations:** 1 Institute of Rare Genetic Disease, PRG S&Tech Co., Ltd., Busan 46274, Republic of Korea; thdud2971@naver.com (S.P.); taegyun0728@prgst.com (T.-G.W.); 2 Department of Molecular Biology, College of Natural Science, Pusan National University, Busan 46241, Republic of Korea; rosa.somi.kang@hanmail.net; 3 Institute of Systems Biology, Pusan National University, Busan 46241, Republic of Korea

**Keywords:** neurofibromatosis type 1, NF2-SWN, selumetinib, bevacizumab, brigatinib, PRG-N-01, AR-42

## Abstract

Neurofibromatosis type 1 (NF1) and NF2-related schwannomatosis (NF2-SWN) are major genetic tumor predisposition syndromes characterized by progressive, often debilitating neoplasms of the peripheral and central nervous systems. Over the past five years, substantial advances in molecular genetics, signaling biology, and targeted therapeutic development have reshaped diagnostic and management paradigms for both disorders. This Perspective synthesizes recent developments, including gene-based reclassification, emergence of MEK inhibitor therapy in NF1, renewed evaluation of bevacizumab and kinase-pathway inhibitor brigatinib, the discovery of a novel TβR1-RKIP pathogenic axis, and a brain-penetrant HDAC inhibitor in NF2-SWN. These insights highlight a shift toward precision-medicine strategies and mechanistically driven therapies poised to redefine future clinical care.

## 1. Introduction

Neurofibromatosis type 1 (NF1) and NF2-related schwannomatosis (NF2-SWN) are lifelong tumor predisposition syndromes that impose cumulative neurological morbidity through multiple, anatomically distributed neoplasms affecting the peripheral and central nervous systems [1,2,3,4]. NF1 and NF2-SWN are distinct hereditary tumor predisposition disorders caused by mutations in different tumor suppressor genes. NF1 results from loss-of-function variants in the NF1 gene on chromosome 17, which encodes neurofibromin, and is characterized by widespread development of neurofibromas, pigmented skin lesions (café-au-lait spots), skeletal abnormalities, and a range of other systemic manifestations beginning in childhood. In contrast, NF2-SWN arises from pathogenic variants in the NF2 gene on chromosome 22, encoding the tumor suppressor merlin; its hallmark is the development of bilateral vestibular schwannomas, along with meningiomas, ependymomas, and other Schwann cell-derived tumors, typically presenting in late adolescence or early adulthood. While both conditions predispose to benign nervous system tumors, their tumor types, affected tissues, clinical trajectories, and underlying molecular etiologies are distinct, reflecting separable pathogenic mechanisms and implications for diagnosis and management [1,2,3,4].

## 2. Reframing NF1 and NF2-SWN as Genotype-Informed and Function-Centered Disorders

Recent updates to the diagnostic criteria for NF1 and the reclassification of NF2 syndrome into NF2-SWN represent a major turning point in neurofibromatosis care [5,6]. For NF1, the formal incorporation of germline NF1 variant testing, the recognition of mosaic presentations, and the explicit delineation of SPRED1 gene-associated Legius syndrome have significantly improved diagnostic clarity [5]. These refinements reduce reliance on late-manifesting clinical features in young children, allowing diagnosis and therefore surveillance and early intervention to occur earlier in the disease course. This is particularly important for preventing delayed recognition of plexiform neurofibromas, optic pathway gliomas, or skeletal abnormalities. Historically, management relied heavily on surgery and radiotherapy, supplemented by symptom-directed supportive care [5,7]. Over the last several years, however, advances in molecular classification, genotype-informed counseling, and targeted systemic therapies have begun to shift NF care toward a longitudinal model in which the timing, sequencing, and mechanism increasingly determine clinical decisions [8,9,10,11,12]. For example, the early identification of children with NF1 and high plexiform neurofibroma burden allows the timely introduction of MEK inhibition alongside physical therapy, pain management, and scoliosis surveillance, rather than waiting for irreversible structural or neurologic complications [10,13,14,15,16].

This transition is most visible in NF1, where MEK inhibition has established a successful therapeutic baseline for symptomatic, inoperable plexiform neurofibromas and has expanded expectations beyond debulking toward sustained tumor control and functional improvement [13,14,15].

Similarly, in NF2-SWN, the 2022 consensus introduced a gene-centered classification system that distinguishes NF2-, SMARCB1-, LZTR1-, and chromosome 22q-associated schwannomatoses. The recognition of NF2, LZTR1, or SMARCB1 variants informs expectations regarding vestibular schwannoma, meningioma, and spinal tumor trajectories and helps prioritize hearing-preservation strategies, visual pathway monitoring, and spinal stability assessment [6,7,17]. This represents a major departure from the former umbrella term “NF2,” which grouped biologically distinct tumor predisposition syndromes into a single category. The updated schema recognizes differences in tumor distribution, age at onset, risk of meningiomas or spinal tumors, and inheritance patterns, enabling more precise prognostication.

Until now, anti-VEGF therapy and multi-kinase inhibition have shown meaningful radiographic and clinical benefit in subsets of NF2-SWN patients, supporting a broader role for systemic treatment within individualized algorithms that prioritize hearing preservation, cranial nerve function, spinal stability, and quality of life [18,19,20,21]. At the same time, the field is moving beyond pathway suppression alone: Mechanism-driven strategies that target merlin-loss-associated signaling consequences and tumor cell state are emerging, including approaches aimed at defined protein interactions, stress-responsive signaling nodes, and epigenetic dependencies [8,22,23]. These efforts raise the possibility that future systemic therapies may not only control tumor growth but also reshape the natural history by reducing the need for repeated cranial and spinal procedures.

Looking ahead, gene- and cell-based approaches represent an aspirational frontier for both NF1 and NF2-SWN. Strategies under exploration include gene replacement or restoration of NF1 or merlin function, allele-specific targeting of pathogenic variants, and genome- or epigenome-editing concepts designed to correct upstream drivers rather than downstream pathways [10,11,12,16].

In this Perspective, we synthesize the therapeutic and conceptual advances shaping this new era, emphasizing how mechanism-based agents and heterogeneity-informed frameworks are beginning to redefine NF1 and NF2-SWN management as coordinated, genotype-informed, and function-centered care across the patient lifespan.

## 3. NF1: MEK Inhibition as the First Mature Era of Molecular Therapy

The advent of MEK inhibitors, most notably selumetinib and mirdametinib, marks the first truly transformative molecular therapy for NF1-associated plexiform neurofibromas (PNs) [13,14,15]. Selumetinib, validated through the pivotal SPRINT trial (NCT01362803: AZD6244 Hydrogen Sulfate for Children with Nervous System Tumors), became the first FDA-approved systemic therapy for NF1-PN, supported by the landmark SPRINT Phase II trial, demonstrating durable tumor shrinkage, functional improvement, and pain reduction in children with inoperable PNs [13]. Subsequent long-term follow-up studies validated the consistency, durability, and safety of MAPK pathway suppression across broader pediatric and adolescent populations [15]. For adults and adolescents, mirdametinib (NCT03962543: MEK Inhibitor Mirdametinib (PD-0325901) in Patients with Neurofibromatosis Type 1 Associated Plexiform Neurofibromas) further expanded the therapeutic landscape, with clinical trials reporting significant reductions in PN volume, improvement in patient-reported outcomes, and manageable toxicity profiles [14,15]. These successes collectively marked the transition of NF1 management from surgical debulking alone to evidence-based pharmacologic intervention, fundamentally altering treatment expectations for patients with PNs. However, long-term surveillance emphasizes several emerging challenges, including drug resistance, incomplete durability of response, and potential endocrine or developmental complications during prolonged MAPK inhibition [15,16]. These considerations have catalyzed active exploration into combination regimens that pair MEK inhibition with mTOR modulation, immune-targeted approaches, or adjunctive surgery for refractory or complex lesions [10,16]. Concurrently, advances in multi-omic, single-cell, and spatial transcriptomic profiling of NF1-associated PNs have provided new insights into intratumoral complexity. Studies now reveal intricate interactions between neoplastic Schwann cell subpopulations, immune infiltrates, and fibroblastic stromal components, alongside the identification of molecular programs such as secreted phosphoprotein 1 (SPP1) signaling that may function as modifier loci, progression markers, or predictors of therapeutic response [9,12]. These findings promise to refine patient selection for MEK inhibitor therapy and guide the design of next-generation therapeutic strategies.

## 4. NF2-Related Schwannomatosis: From VEGF Blockade to Multi-Pathway Targeting

**Figure 1:**
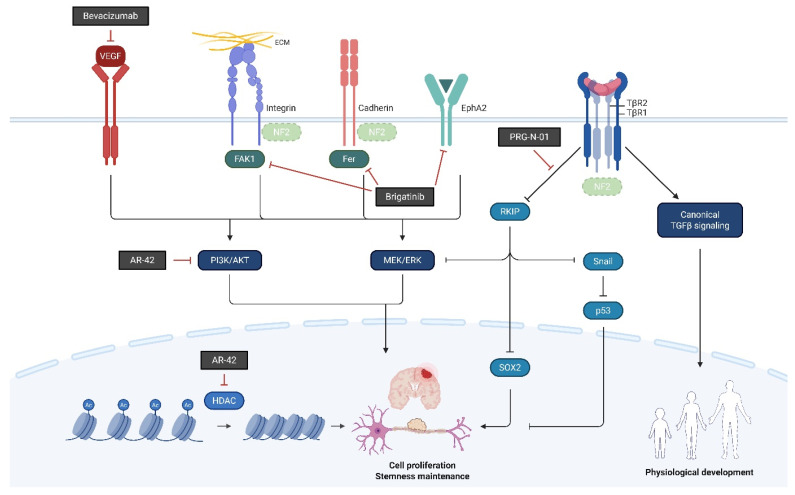
Therapeutic agents in development for NF2-related schwannomatosis (NF2-SWN) and their molecular targets. This scheme illustrates representative therapeutic agents investigated for the treatment of NF2-SWN and their primary molecular targets: Bevacizumab inhibits VEGF-mediated angiogenesis, while brigatinib functions as a multi-kinase inhibitor with activity against receptor and non-receptor kinases involved in tumor-promoting signals. PRG-N-01 represents an emerging therapeutic approach that modulates aberrant TβR1-mediated noncanonical TGFβ signaling, resulting in induction of RKIP, a tumor suppressor. AR-42, an HDAC inhibitor, exerts epigenetic regulation associated with tumor growth suppression and reduction in AKT hyperactivation. Collectively, these agents highlight diverse therapeutic strategies under investigation for NF2-SWN (Created in BioRender. So-young, P. https://BioRender.com/fdpcxbm (accessed on 1 March 2026)).

In NF2-SWN, recent advances in molecularly targeted and epigenetic therapies have identified several candidate agents, including bevacizumab, brigatinib, PRG-N-01, and AR-42, that demonstrate potential therapeutic activity. This section provides an overview of these agents (Figure 1, Table 1).

### 4.1. Bevacizumab: Effective but Not Disease-Modifying

Bevacizumab remains the most consistently effective systemic therapy for NF2-related vestibular schwannomas, with prospective trials demonstrating hearing improvement, tumor regression, or radiographic stabilization in 30–60% of patients [18,20,25]. Notably, its functional benefits may extend beyond volumetric reduction, including decreased edema, vascular permeability, and cochlear nerve compression [20,26]. Several limitations have emerged through long-term follow-up. Tachyphylaxis, progressive loss of effect, is increasingly recognized during prolonged continuous administration [26]. Adverse effects such as hypertension, proteinuria, impaired wound healing, menstrual irregularities, and rare thromboembolic complications hinder long-term use [18,25,26,27]. Pediatric patients show comparable initial responses yet may exhibit unique vulnerabilities related to growth and endocrine systems during chronic VEGF blockade [26]. Efforts to optimize bevacizumab use, including intermittent dosing, low-dose maintenance therapy, and switching strategies at early signs of resistance, are ongoing [20,22,26,28]. Nevertheless, VEGF inhibition does not address the fundamental molecular consequences of merlin loss and therefore is not considered disease-modifying [17,28]. These limitations have highlighted the need for more mechanistically grounded therapeutics, including multi-kinase inhibitors such as brigatinib.

### 4.2. Brigatinib: A Leading Multi-Kinase Candidate

Brigatinib has emerged as a leading multi-kinase therapeutic candidate for NF2-related schwannomatosis (NF2-SWN). Preclinical work from the Synodos for NF2 consortium identified brigatinib as a potent inhibitor of NF2-deficient meningioma and schwannoma growth, acting through inhibition of multiple tyrosine kinases including EphA2, Fer, and focal adhesion kinase 1 (FAK1) rather than ALK [19]. These targets converge on signaling axes such as PI3K/AKT, ERK, and mTOR, which are known to be hyperactivated in merlin-deficient Schwann and meningeal cells [29,30]. Together, these data position brigatinib among the most promising next-generation systemic therapeutics for NF2-SWN [8]. On the basis of these preclinical findings, the INTUITT-NF2 phase II trial (NCT04374305: Innovative Trial for Understanding the Impact of Targeted Therapies in NF2-Related Schwannomatosis) provided the first prospective clinical validation of brigatinib, showing radiographic responses across multiple NF2-associated tumor types, improvements in hearing or pain in subsets of patients, and an overall favorable safety profile without grade 4–5 toxicity [21,31]. In this study, oral brigatinib (180 mg once daily after lead-in) produced radiographic responses across multiple tumor types including vestibular and non-vestibular schwannomas, meningiomas, and ependymomas with associated clinical benefit in a heavily pretreated cohort, including improvements in hearing or pain in a subset of patients and no grade 4–5 treatment-related adverse events. Despite its strengths, the trial has limitations, such as the lack of a placebo control and its restriction to patients at specialty centers, potentially limiting generalizability. Moreover, the small sample size resulted in broad confidence intervals for some outcomes. Importantly, the trial reported no severe treatment-related adverse events, and most adverse events (grade 3) were generally manageable, suggesting that Brigatinib has a more favorable safety profile compared to Bevacizumab, which is known for its cumulative toxicity. But in another analysis about the adverse events caused by Brigatinib, the need for retrospective analysis of adverse drug reactions and the establishment of adverse event monitoring systems for long-term treatment for patients were suggested. In summary, this trial provided the first prospective clinical evidence that multi-kinase inhibition with brigatinib can deliver meaningful tumor control and symptom improvement in NF2-SWN. But continued research and development to understand the cause of the disease is essential to improve the lives of those affected by this challenging disease.

### 4.3. Discovery of a Novel RKIP-TGF-β-Recptor1 Pathogenic Axis

A converging body of molecular evidence has recently reshaped our understanding of NF2-associated tumorigenesis by revealing a previously unrecognized signaling cascade centered on merlin deficiency, TGFβ receptor instability and RKIP dysregulation, a pathway now directly targetable by the first-in-class small molecule PRG-N-01. Loss of merlin destabilizes TGF-β-Receptor 2 (TβR2), leading to aberrant membrane dynamics and compensatory hyperactivation of TβR1-mediated noncanonical TGFβ signaling, including MAPK, ERK, and associated oncogenic branches [32]. This imbalance primes Schwann and meningeal cells for pathological signaling amplification, increasing susceptibility to microenvironmental or mechanical stimuli. Mechanistic studies further demonstrate that merlin loss promotes snail-driven suppression of p53, lowering the apoptotic threshold and enabling EMT-like cellular reprogramming [33]. In parallel, NF2 deficiency disrupts RKIP homeostasis: merlin-deficient cells show RKIP degradation, which in turn enhances oncogenic MAPK flux and impairs differentiation pathways [23,34]. Building on these mechanistic foundations, the neuro-oncology report describing PRG-N-01 provides a unifying model in which physical or mechanical stress triggers aberrant engagement between TβR1 and RKIP, resulting in RKIP phosphorylation, destabilization, and degradation, thereby removing a key inhibitory brake on MAPK signaling [35]. Importantly, the study demonstrates that PRG-N-01 selectively blocks the pathological TβR1–RKIP interaction, restores RKIP stability, and suppresses downstream MAPK hyperactivation [35]. Beyond mechanistic specificity, PRG-N-01 demonstrates several pharmacologic properties that support its therapeutic viability. Unlike ATP-competitive kinase inhibitors, PRG-N-01 does not broadly inhibit TβR1 catalytic activity or canonical SMAD phosphorylation. Instead, it selectively interferes with the stress-induced protein–protein interaction between TβR1 and RKIP without affecting basal TGFβ signaling [35]. This confers pathway specificity and reduces the risk of off-target effects commonly associated with TGFβ pathway inhibitors. Structural modeling and mutagenesis analyses indicate that PRG-N-01 binds to a defined pocket at the RKIP-TβR1 interaction surface, conferring both chemical tractability and medicinal-chemistry expandability [35]. This establishes PRG-N-01 as a druggable PPI (protein–protein interaction) inhibitor. Restoration of RKIP is itself therapeutically meaningful: recent work shows that RKIP induction reduces SOX2 stability and promotes the differentiation of NF2-deficient tumor cells, highlighting RKIP as a gatekeeper of differentiation state and tumor plasticity [23]. By stabilizing RKIP, PRG-N-01 effectively reinstates this tumor-suppressive axis. Consistent with these mechanistic insights, PRG-N-01 induces morphologic and transcriptional differentiation in HEI-193 schwannoma cells and demonstrates significant anti-tumor activity in vivo, including reduced tumor volume and proliferative index in multiple NF2-associated tumor models [35]. Taken together, these findings define a new RKIP-merlin–TβR2 pathway in Mesothelium, Schwann and Meningeal cells. The TβR1–RKIP axis was defined as a central pathogenic pathway in NF2-related schwannomatosis (merlin-deficient condition), integrating aberrant TGFβ receptor dynamics, MAPK pathway overload, snail-dependent p53 suppression, and missing control of differentiation in RKIP deficiency. Building on this strong mechanistic and preclinical foundation, PRG-N-01 has now advanced into clinical evaluation. The Ministry of Food and Drug Safety (MFDS) of the Republic of Korea approved the initiation of a first-in-human phase 1/2 clinical trial (KCT0009520: A Study Evaluating the Safety and Efficacy of PRG-N-01 in Patients with Neurofibromatosis Type II), which began in May 2024 and is scheduled to conclude in 2026. Notably, at the 2025 Children’s Tumor Foundation (CTF) NF Conference (Washington, DC, USA), interim data from this ongoing trial demonstrated positive therapeutic signals with no treatment-related adverse events in the Clinical Platforms section (https://www.ctf.org/wp-content/uploads/2025/06/25_NFConferenceAbstractBook_web.pdf?eu=, accessed on 17 March 2026), providing early clinical validation of PRG-N-01’s safety and translational potential in patients with NF2-related schwannomatosis.

### 4.4. AR-42 (REC-2282): A Brain-Penetrant Pan-HDAC Inhibitor

AR-42 (REC-2282) is an orally bioavailable pan-HDAC inhibitor incorporating a hydroxamate-tethered phenylbutyrate scaffold that drives histone H3/H4 acetylation, tubulin acetylation, inhibition of PI3K/AKT signaling, and induction of G2 arrest and caspase-dependent apoptosis [36,37,38,39,40,41]. Through these epigenetic and signaling mechanisms, AR-42 demonstrates broad antitumor activity across multiple hematologic and solid tumors [42,43,44,45,46]. Preclinical studies have shown notable efficacy in B-cell malignancies, including CLL, mantle cell lymphoma, ALL or lymphoma, multiple myeloma, and Burkitt lymphoma, with AR-42 demonstrating superior antitumor activity compared with other HDAC inhibitors, such as vorinostat, in vivo [38,39,42]. Importantly for NF2-related disease, AR-42 shows potent activity against vestibular schwannomas and NF2-deficient meningiomas [36,37,46,47]. In these systems, AR-42 reverses aberrant epigenetic states, dampens PI3K/AKT activation, and triggers apoptosis in merlin-deficient tumor cells. These findings highlight HDAC inhibition as a mechanistically distinct approach that complements kinase- and receptor-directed strategies under evaluation in NF2-related schwannomatosis. A major pharmacologic strength of AR-42 is its ability to penetrate the blood–brain barrier. Rodent pharmacokinetic studies show that AR-42 achieves therapeutically relevant CNS tissue concentrations with sustained exposure following oral dosing [48]. This property distinguishes AR-42 from several HDAC inhibitors with limited intracranial distribution and supports its potential utility for tumors arising in the nervous system, including schwannomas and meningiomas. Clinically, a phase I study in multiple myeloma and T- and B-cell lymphomas established the maximum tolerated dose as 40 mg three times weekly, with durable responses in individual patients and an overall favorable safety profile [49]. These data provided the foundation for expanding AR-42 development into rare solid tumors, including NF2-related schwannomatosis. Based on its CNS penetrance and strong preclinical efficacy in NF2 models, AR-42 (REC-2282) advanced into dedicated NF2 clinical evaluation, including through the REC-2282-201 study (NCT05130866: Efficacy and Safety of REC-2282 in Patients with Progressive Neurofibromatosis Type 2 Mutated Meningiomas), originally designed to enroll patients through 2027. Despite the absence of safety issues or futility signals, the trial was terminated early by the sponsor due to internal pipeline reprioritization. This outcome illustrates the difficulties of sustaining drug development in ultra-rare tumors, even when mechanistic rationale and preclinical evidence are compelling. Collectively, AR-42 remains one of the most extensively studied epigenetic agents relevant to NF2. Its broad antitumor effects, CNS access, and confirmed human tolerability underscore the potential value of HDAC-directed strategies in NF2-related schwannomatosis, and support the continued exploration of epigenetic therapeutics in this setting.

## 5. Concluding Remarks: Toward Disease Modification

Neurofibromatosis type 1 (NF1) and NF2-related schwannomatosis (NF2-SWN) are entering a period of conceptual and therapeutic redefinition [5,6]. In NF1, MEK inhibition represents the first mature era of mechanism-based therapy, demonstrating that sustained pathway modulation can alter tumor behavior and clinical outcomes [13,14]. These advances have reframed expectations for patients with plexiform neurofibromas and catalyzed broader efforts to refine patient selection, optimize long-term safety, and develop combination or next-generation approaches informed by tumor heterogeneity. In NF2-SWN, experience with anti-VEGF therapy and multi-kinase inhibition has similarly shown that systemic therapy can yield meaningful clinical benefit. At the same time, these experiences highlight an important limitation: many currently available agents do not directly address the molecular consequences of merlin loss. This recognition has accelerated interest in therapeutics targeting defined signaling nodes, protein–protein interactions, and epigenetic dependencies that shape NF2-deficient tumor biology [23,35].

Despite these advances, several critical knowledge gaps remain. The biological determinants of tumor initiation and progression in both NF1 and NF2-SWN remain incompletely understood, particularly with respect to cellular heterogeneity, microenvironmental influences, and mechanisms of therapeutic resistance. Reliable predictive biomarkers for treatment response are still lacking, and most systemic therapies have been evaluated primarily for a limited subset of manifestations rather than across the full clinical spectrum of disease. In NF1, for example, MEK inhibition represents a major therapeutic milestone for plexiform neurofibromas, yet disease-modifying systemic options remain unavailable for many other NF1-associated manifestations, including optic pathway gliomas, skeletal abnormalities, cognitive features, and malignant transformation risk. Similarly, in NF2-related schwannomatosis, existing systemic agents may provide symptomatic or radiographic benefit in subsets of patients but do not yet offer durable control of tumor predisposition or address the fundamental consequences of merlin loss.

Additional investigational strategies are also being explored. Immune checkpoint blockade, including anti-PD-1 therapies, has shown limited but evolving evidence in NF-associated tumors [24]. Metabolic approaches targeting fatty-acid synthase, such as TVB-2640 (denifanstat), as well as earlier agents including cerulenin, represent efforts to disrupt tumor metabolic dependencies [50]. Epigenetic strategies beyond AR-42, including the HDAC inhibitor vorinostat, have also been investigated in preclinical and early clinical settings, although clinical efficacy remains to be established [42].

Addressing these challenges will require several research priorities. First, deeper molecular characterization of NF-associated tumors using single-cell and spatial profiling will be essential to define actionable vulnerabilities and clarify the cellular states that drive tumor growth or treatment resistance [7,17]. Second, the development of robust biomarkers—including circulating markers, imaging-based indicators, and functional endpoints—will be necessary to guide patient selection and monitor therapeutic response. Third, systematic evaluation of rational combination strategies targeting complementary pathways may help overcome the limitations of single-agent approaches and improve the durability of responses.

At the clinical level, the effective management of neurofibromatosis will increasingly depend on integrated therapeutic frameworks rather than the replacement of established modalities. Systemic therapies are likely to be deployed alongside surgery, radiosurgery, rehabilitation, and structured surveillance within coordinated multidisciplinary programs [10,16].

Looking forward, gene- and cell-based therapeutic strategies represent an aspirational direction for addressing the root causes of tumor predisposition in neurofibromatosis [10,11,12,16]. Although these approaches remain in early stages of development, successful translation could ultimately shift treatment paradigms from symptomatic management toward durable disease modification.

## Figures and Tables

**Table 1:** NF1/NF2 therapeutic agents (mechanism, phase, location, safety, efficacy).

Agent	Structural Formula	Indication	Mechanism of Action	Clinical Trial Phase (Representative)	Clinical Location (As Reported)	Side-Effects Reported	Efficacy Signals Reported
Bevacizumab	Antibody	NF2/NF2-SWN: vestibular schwannoma (VS) and meningiomas	Anti-VEGF monoclonal antibody reduces tumor vascular permeability/angiogenesis	NCT01207687; NCT01767792; NCT01125046: multiple Phase 2 clinical studies	Multicenter/specialty NF2 centers (registry + academic reports; locations vary by protocol)	Typical bevacizumab risks in NF2 series include hypertension, proteinuria, bleeding/thromboembolism, impaired wound healing (grade/severity depends on regimen)	Hearing and tumor effects reported in NF2-VS cohorts (e.g., hearing improvement and tumor shrinkage in subsets; maintenance strategies studied)
Brigatinib	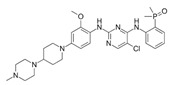	NF2-SWN: VS, non-VS schwannomas, meningiomas, ependymomas	Multi-kinase inhibitor (clinically known as an ALK inhibitor; trial rationale based on activity in NF2-driven tumors)	Phase 2 platform/basket trial (INTUITT-NF2; NCT04374305)	Multicenter (7 locations in U.S.) (platform trial)	No grade 4/5 treatment-related AEs reported in NEJM INTUITT-NF2 publication; overall tolerability described as favorable in that cohort	After median follow-up (~10.4 mo): radiographic response 10% (target) and 23% (overall); hearing improvement in 35%; greatest benefit in meningiomas/non-VS schwannomas
PRG-N-01 (Trineumin)	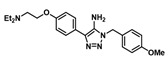	NF2/NF2-SWN (investigational therapy being studied in adults)	Mechanism-based NF2 therapy targeting TbR1-RKIP pathological interaction	KCT0009520: Phase1/2a (dose/early efficacy learning objectives)	Asan medical center in Korea	Public trial safety/AE profile not yet mature in peer-reviewed form (as of available public sources); preclinical tox/ADME described	Public sources indicate an ongoing trial in Korea; current peer-reviewed studies focus on preclinical druggability rather than definitive clinical efficacy
REC-2282 (formerly AR-42)	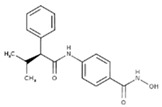	NF2-mutated progressive meningiomas	Pan-histone deacetylase (HDAC) inhibitor inducing cell-cycle arrest, apoptosis, and AKT suppression in NF2-deficient cells	NCT05130866: Phase 2/3 randomized multicenter trial	Multicenter study (U.S. and international sites)	Safety under evaluation; HDAC inhibitors commonly associated with fatigue, cytopenia, gastrointestinal toxicity	Primary endpoint: progression-free survival (PFS); efficacy results not yet reported (trial ongoing)
Selumetinib	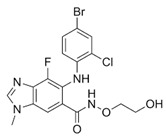	NF1: plexiform neurofibroma (PN); also explored in cutaneous neurofibromas and other NF1 manifestations	MEK1/2 inhibitor (MAPK pathway suppression downstream of RAS)	NCT01362803: Pivotal phase 2-Pediatric PN and other NF1 studies ongoing/updated in later reports	Typically multicenter (NCI-led and international academic networks depending on protocol)	Common AEs: GI symptoms, acneiform rash, paronychia, CK elevation; less common cardiac and ocular toxicity	Pediatric PN: meaningful tumor reduction and symptom benefit; adult NF1 PN: volume reduction and pain improvement reported
Mirdametinib	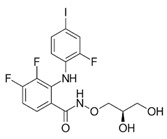	NF1: symptomatic plexiform neurofibroma	MEK1/2 inhibitor (targeting the RAS–MAPK pathway.	NCT03962543: Pivotal phase 2b-adults + children PN	Multicenter	Common treatment-related AEs: adults—dermatitis acneiform, diarrhea, nausea; children—dermatitis acneiform, diarrhea, paronychia	Confirmed ORR (≥20% PN reduction): 41% adults, 52% children; median PN change ~−41% to −42%; pain/HRQoL improved
Mirdametinib + Vorinostat	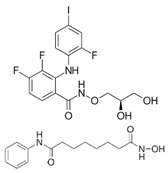	NF1-associated malignant peripheral nerve sheath tumor (MPNST) with H3K27 trimethylation deficiency (PRC2-deficient tumors)	Mirdametinib (MEK1/2 inhibitor) + Vorinostat (HDAC inhibitor): dual targeting of MAPK signaling and epigenetic dysregulation.	NCT06693284: Phase 0/early phase (“window-of-opportunity” trial)	University of Minnesota (single-center study)	Primary objective: safety/tolerability and pharmacodynamic tumor effects; toxicity still under investigation.	Evaluates tumor response and pharmacodynamics after 28-day pre-op treatment (imaging/biopsy before standard therapy); definitive efficacy not yet reported
Anti-PD-1 (e.g., pembrolizumab)	Antibody	NF1-associated metastatic malignant peripheral nerve sheath tumor (MPNST) with CD274/PD-L1 amplification	Immune checkpoint blockade: PD-1 inhibition → restores anti-tumor T-cell activity	Clinical case report (Özdemir et al., 2019, JCO Precision Oncology) [24]	Switzerland (single-patient clinical observation)	Immune-related adverse events typical of PD-1 blockade (e.g., dermatitis, colitis, endocrinopathies) were manageable; no severe unexpected toxicity reported in this case	Deep, durable response in metastatic NF1-MPNST, associated with PD-L1 amplification/high expression, suggesting a predictive biomarker

## Data Availability

No new data were created or analyzed in this study. Data sharing is not applicable to this article.
